# Suicide Mortality Trends in Four Provinces of Iran with the Highest Mortality, from 2006-2016 

**Published:** 2017-06-14

**Authors:** Hajar Nazari Kangavari, Ahmad Shojaei, Seyed Saeed Hashemi Nazari

**Affiliations:** ^1^ Department of Epidemiology, School of Public Health, Shahid Beheshti University of Medical Sciences, Tehran, Iran; ^2^ Legal Medicine Research Center, Iranian Legal Medicine Organization, Tehran, Iran; ^3^Safety Promotion and Injury Prevention Research Center, Department of Epidemiology, School of Public Health, Shahid Beheshti University of Medical Sciences, Tehran, Iran

**Keywords:** Suicide, Mortality, Interrupted Time series, Iran

## Abstract

**Background:** Suicide is a major cause of unnatural deaths in the world. Its incidence is higher in western provinces of Iran. So far, there has not been any time series analysis of suicide in western provinces. The purpose of this study was to analyze suicide mortality data from 2006 to 2016 as well as to forecast the number of suicides for 2017 in four provinces of Iran (Ilam, Kermanshah and Lorestan and Kohgiluyeh and Boyer-Ahmad).

**Study design:** Descriptive-analytic study.

**Methods:** Data were analyzed by time series analysis using R software. Three automatic methods (Auto.arima, ETS (Error Transitional Seasonality) and time series linear model (TSLM)) were fitted on the data. The best model after cross validation according to the mean absolute error measure was selected for forecasting.

**Results:** Totally, 7004 suicidal deaths occurred of which, 4259 were male and 2745 were female. The mean age of the study population was (32.05 ± 15.48 yr). Hanging and self-immolation were the most frequent types of suicide in men and women, respectively. The maximum and minimum number of suicides was occurred in July and August as well as January respectively.

**Conclusions:** It is suggested that intervention measures should be designed in order to decrease the suicide rate particularly in the age group of 15-29 yr, and implemented as a pilot study, especially in these four provinces of Iran, which have a relatively high suicide rate.

## Introduction

Suicide is a major cause of unnatural deaths, fifteenth leading cause of death and responsible for 1.4% of all deaths in the world. According WHO in 2014, about 804,000 suicidal deaths occurred in 2012, about 75% of which happened in developing and low-income countries^1^.

Epidemiological distribution of suicide depends on a complex set of factors, including demographic, cultural, economic and social factors, time, location, availability of equipment and so on. However, most researchers have cited factors such as gender, age, religion, marital status, occupation, race, climatic conditions, physical and mental conditions as contributing factors. Among the methods of suicide, hanging, poisoning and suicide by firearms are the most common^1-5^. Of course, the choice of suicide method is often different based on demographic groups^1, 6, 7^. Different spatial and temporal patterns of suicide in different areas have been accessed. Different suicide rates exist in different geographic areas in each state of Iran^1, 8-11^.

The results of investigations in legal medical organization showed that the suicide rate in Iran is about five cases per 100,000 population^6^. Although the rate is lower than most countries in the world, especially Western countries, but it is higher than Middle East region average^1^. This situation is not uniform throughout Iran and some provinces are in critical condition. The alarming growth of 11.5% of successful suicides in 2013 compared to 2012 indicates a need for further studies in this field^12^. Significant differences in suicide methods and demographic variations in suicide rate in terms of gender, age and other factors among provinces, show different patterns of suicide in the country and the need to look at the regional social damage. Because the incidence of suicide was higher in western provinces of Iran. So far, there has not been any time series analysis of suicide in western provinces. Assessing the demographic characteristics and trend of suicide rate in the past years and forecasting it enables us to make the appropriate planning for prevention and control.

This study aimed to analyze time series data on suicide mortality from 2006 to 2016 in four provinces of Iran (Ilam, Kermanshah and Lorestan and Kohgiluyeh and Boyer-Ahmad) that had the highest suicide rate in 2016 and forecast suicide for the coming year in these four provinces.

## Methods

This descriptive-analytic study was conducted in four provinces of Iran: (Ilam, Kermanshah, Lorestan and Kohgiluyeh and Boyer-Ahmad) which had the highest suicide rate in 2016. From a geographical point of view, Ilam, Kermanshah and Lorestan provinces are located in the West and Kohgiluyeh and Boyer-Ahmad is located in southwestern of Iran. According to the Iranian's law, all the suspicious deaths should be referred to legal medicine organization. Death certificate for these kinds of deaths should be issued just by this organization. So it is expected that almost all suicides occurred in the country, be recorded by this organization and the suicide data of this organization be the most complete source for suicide mortality data. Hence, for this analysis, data on demographic characteristics of each suicide death such as gender, age, month, province and methods of suicide and other information was received from legal medical organization.

After cleaning the data, 10-yr suicide rates (per 100,000 populations) in four provinces of Iran were calculated. For time series analysis the frequency (count) of suicide deaths in each month for a period of 120 months, from April 2006 to March 2016 were entered separately for each province in R software (version 3.3.2) and descriptive and time series analysis of the data were performed. We used Fit.AR, tseries, forecast and fpp packages. Age variable was divided into five categories (5-14, 15-29, 30-49, 50-69 and ≥70 yr old) and methods of suicide were grouped into four categories (hanging, Immolation, poisoning and other methods). In order to do time series analysis, Box-Jenkins methods was used. In time series analysis by Box-Jenkins method, depending on the structure of data, ARIMA or SARIMA models are used to predict the future. Drawing plot of series is the first step in modeling a time series analysis. Useful information about the nature of data can be obtained from time series plots. Time-series plot helps to identify trends, seasonality and non-stationarity of variance and mean^13^.

First, the non-stationarity at variance was evaluated using the Box-Cox test and if it was indicated, the data were transformed. Next, the non-stationarity at mean was checked using the Kwiatkowski-Phillips-Schmidt-Shin (KPSS) test. After reviewing the series in terms of the stationarity in variance and mean, autocorrelation function and partial autocorrelation function curves were plotted on the raw or transformed data. In the next step of analyze, the three auto-prediction methods; Auto-ARIMA, Error Trend or (Transitional) and Seasonality (ETS) and time series linear model (TSLM) Method were fitted to the data. In these three methods by default, non-stationarity of the mean is evaluated via unit root tests such as Augmented Dickey-Fuller (ADF) Kwiatkowski-Phillips-Schmidt-Shin (KPSS) and Phillips-Perron tests ^14, 15^. Auto-ARIMA method fits different ARIMA models to univariate time series data, selects the best ARIMA model according to either AIC, AICc or BIC value, and conducts a search over possible models within the order of the constraints provided. ETS uses exponential smoothing for all three of error, trend and seasonal terms; TSLM is used to fit linear models to time series including trend and seasonality components^16^.

Then we used k-fold cross validation (C.V) method for assessing how the results of a statistical analysis would generalize to an independent data set. In this method, data is repeatedly divided into two sections, (training and testing dataset). Cross validation method, compare the best-selected model of each function according to the Mean Absolute Error (MAE) index and chose the best valid model for prediction. Cross Validation is a statistical method that produces an estimate of forecast witch has less bias. It is a technique to assess the generalizability of the results obtained based on a set of data to another set of data. We used the first 40 data points as the training set and then in an iterative way added each data point to the test set and after remodeling and reforecasting we calculated the MAE and then averages of all of the MAE. The model, which produced the minimum MAE, was the chosen model. The predictions are reported according to this model^17-19^.

## Results

From 2006 to 2016, 7004 successful suicides were occurred. Among them, 4259 (61%) were male and 2745 (39%) were female. The ratio of male to female was (1.5). The mean age was 32.0 ± 15.4 with minimum and of 5-114 year.

**Figure 1:**
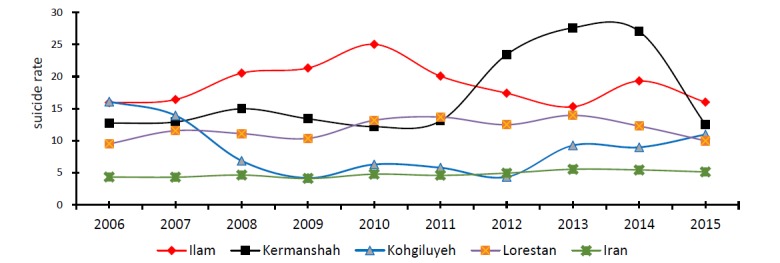
Comparison of 10-year suicide rate (per 100,000 populations) in four provinces of Iran

The lowest and highest age groups were respectively (5-14) and (15-29) yr old. Most suicides had occurred by the method of hanging (35.3%) and self-immolation method (23.9) in men and women, respectively as the most frequent method. The highest and the lowest suicides rate happened in July and August (9.9%), and in January (6.8%), respectively. Mean suicide rate for 10 years of study for Ilam, Kermanshah, Kohgiluyeh and Boyer-Ahmad and Lorestan was 18.7, 16.9, 8.6 and 11.8, respectively (Table 1 and Figure 1).

**Table 1:** The epidemiological Properties of suicide lead to death in 4 provinces were reported from Legal Medicine Organization since 2006 to 2016

**Variables**	**Ilam** **n=1056**		**Kermanshah** **n=3293**		**Kohgiluyeh &** **Boyer-Ahmad** **n=576**		**Lorestan** **n=2079**	
	**Number**	**Percent**	**Number**	**Percent**	**Number**	**Percent**	**Number**	**Percent**
Age groups (yr)								
5-14	11	1.0	84	2.5	22	3.8	24	1.1
15-29	583	55.2	1597	84.5	372	64.5	1266	60.8
30-49	281	26.6	1031	31.3	120	20.8	567	27.2
50-69	115	10.8	344	10.4	33	5.7	170	8.1
≥70	54	5.1	178	5.4	21	3.6	36	1.7
Gender								
Male	565	53.5	2121	64.4	391	67.8	1182	56.8
Female	491	46.5	1172	35.5	185	32.1	897	43.1
Method of suicide								
Hanging	383	36.2	1100	33.4	242	42.0	750	36.0
Self-immolation	370	35.0	752	22.8	60	10.4	494	23.7
Poisoning	137	12.9	576	17.4	37	6.4	511	24.5
Other methods	164	15.5	860	26.1	106	18.4	313	15.0

**Figure 2:**
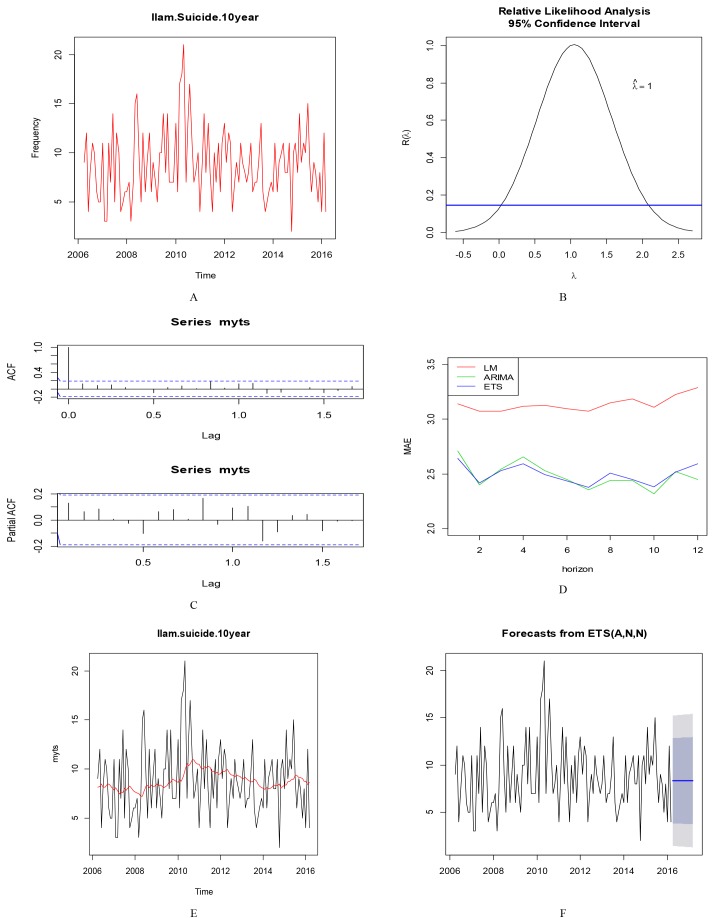
Time series analysis of Ilam province from 2006-2016: (A)Time series plot 10 year of suicide lead to death in Ilam province from 2006-2016; (B) BoxCox graph suicide Ilam province. (C)ACF and PACF in Ilam province from 2006-2016. (D)Comparison of three automatic forecasting methods. (E)Compare proposed models fitted on original data. (F) Forecasting plot from ETS (A, N, N) in Ilam province from 2016-2017.

To perform time series analysis, at first, time series plot of Ilam Province was drawn. The series was stationary for mean and variance. To measure the stationary in terms of variance, fpp package was used. The series was stationarity in terms of variance so Box-Cox transformation was not required for the data. No pattern of auto-regressive and moving average was seen in autocorrelation and partial autocorrelation plots. After fitting all models, ETS method was selected as the best method from three automatically prediction methods using Cross Validation procedure. The parameters of the selected ETS model were (A, N, N) with Smoothing parameters (alpha = 0), Initial state (l = 8.1), sigma (3.5), which denoted that the selected model had just an additive error parameter without trend or seasonal parameter. The corrected version of Akaike information criterion was **(**AICc=882.6). Figure 2 shows time series plot of Ilam Province, BoxCox diagram for checking the stationarity of variance, ACF and PACF plot, comparison of the three methods using C.V, fitted model on original series and the selected forecasting model ets (A, N, N).

**Figure 3:**
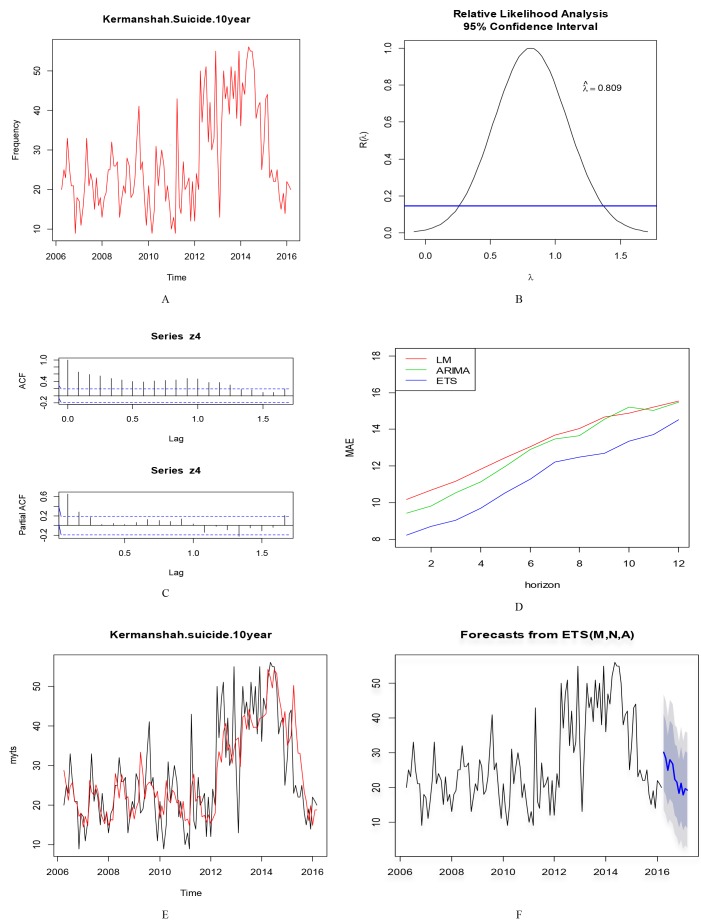
Time series analysis of Kermanshah province from 2006-2016: (A)Time series plot 10 year of suicide lead to death in Kermanshah province from 2006-2016; (B) BoxCox graph suicide Kermanshah province; (C) ACF and PACF in Kermanshah province from 2006-2016; (D) Comparison of three automatic forecasting methods.(E)Compare proposed models fitted on original data.(F) Forecasting plot from ETS (M, N, A) in Kermanshah province from 2016-2017

The time series plot of Kermanshah Province indicated no trend in the data but non-stationary in variance was seen. After performing the BoxCox command, due to the lack of stationary of variance, Box-Cox transformation was performed on the series. ETS was selected as the best method because it had the lowest (MAE) and the ETS (M, N, A) was used to predict. The parameters of the model were (alpha = 0.2) and (gamma = 1e-04), sigma (0.2), Initial state (l=21.8).The corrected version of Akaike information criterion was AICc=1083.2. Series of Kermanshah Province was a series with Multiplicative Error term and the Additive Seasonal component, but trend term was not seen in this series (Figure 3).

**Figure 4:**
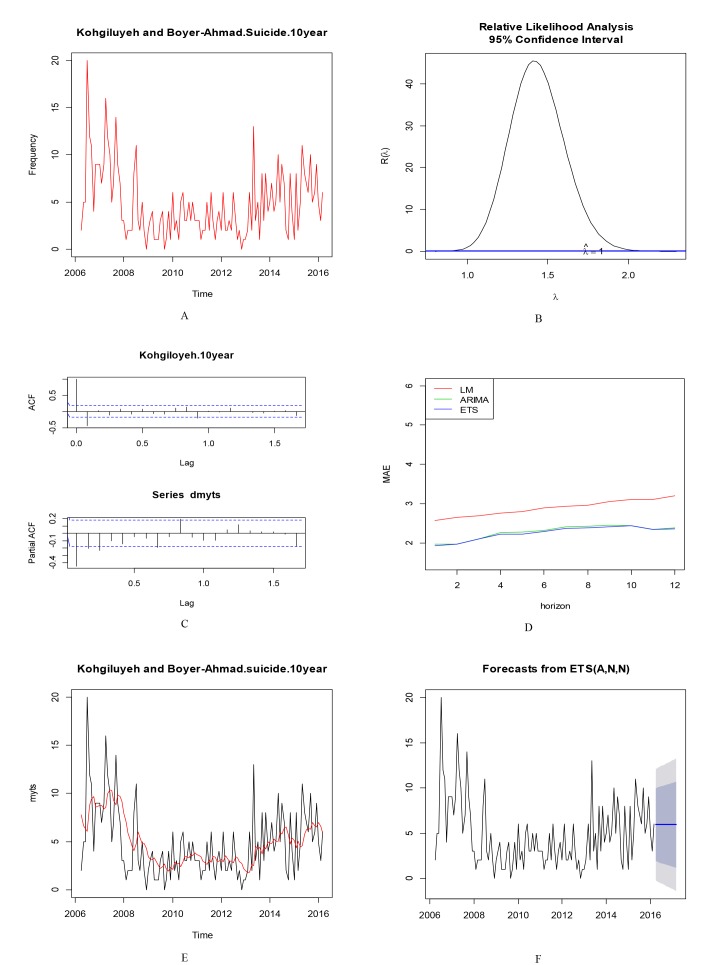
Time series analysis of Kohgiluyeh and Boyer-Ahmad province from 2006-2016.(A)Time series plot 10year of suicide lead to death in Kohgiluyeh and Boyer-Ahmad province from 2006-2016.(B)BoxCox graph suicide Kohgiluyeh and Boyer-Ahmad province.(C) ACF and PACF in Kohgiluyeh and Boyer-Ahmad province from 2006-2016.(D) Comparison of three automatic forecasting methods.(E) Compare proposed models fitted on original data.(F) Forecasting plot from ETS (A, N, N) in Kohgiluyeh and Boyer-Ahmad province from 2016-2017.

There was no trend in the time series plot of Kohgiluyeh and Boyer-Ahmad Province, but nonstationary of variance was seen in the plot. In addition, due to the non-stationary of variance, Box-Cox transformation was performed on the series. ETS method selected to forecast the future values by c.v procedure. The proposed model ETS (A, N, N) was chosen as the best model. The parameters of the model were (alpha = 0.19), sigma (3.1), Initial state (l = 7.7). The corrected version of Akaike information criterion was AICc=856.0. Series had Additive error but seasonality and trend were not seen in the series (Figure 4).

**Figure 5:**
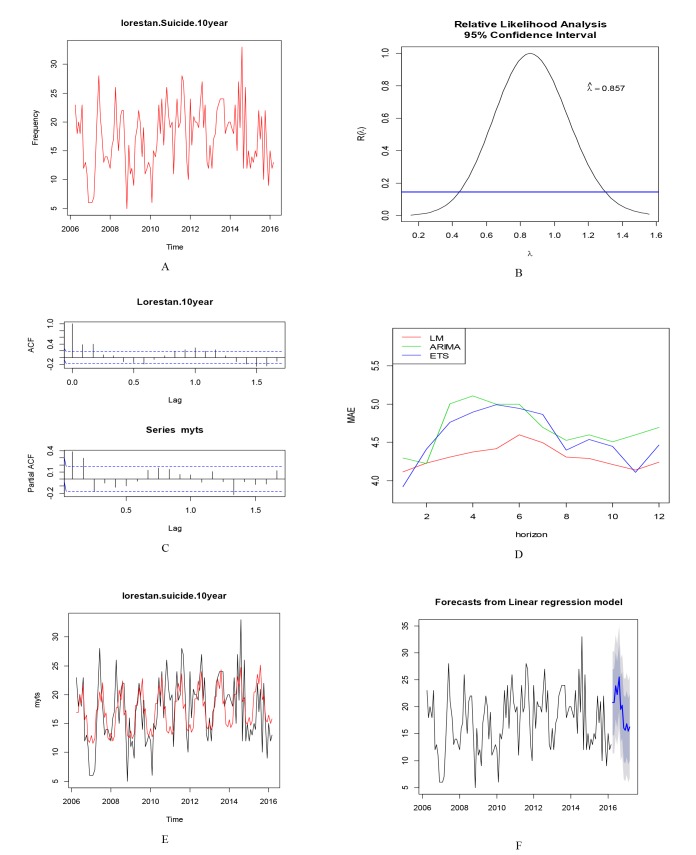
Time series analysis of Lorestan province from 2006-2016. (A)Time series plot 10year of suicide lead to death in Lorestan province from 2006-2016. (B) BoxCox graph suicide Lorestan province.(C)ACF and PACF in Lorestan province from 2006-2016.(D)Comparison of three automatic forecasting methods.(E)Compare proposed models fitted on original data.(F)Forecasting plot from Linear regression model in Lorestan province from 2016-2017.

Time series analysis of Lorestan Province, showed that the mean and variance series were stationary, therefore Box-Cox transformation and differencing were not required for the data. Trend term was not seen in this series. TSLM method was selected as the best method among three methods (Figure 5).

According to the best-fitted models in each province, the forecasts of frequency of suicides in the 12-month period leading to March 2017 in Ilam, Kermanshah, Kohgiluyeh and Boyer-Ahmad and Lorestan were 100, 278, 71 and 233, respectively

## Discussion

The individual and environmental factors are involved in suicide^1^. In Kermanshah Province, depression and sever changes in the mood, lack of interest in work and the lower education level were risk factors affected the occurrence of suicide^20^. In Ilam Province, the occurrence of suicide varied by month and season and factors such as psychological, mental, environmental and social aspects were important on suicide^21, 22^.

From the point of the gender, the frequency of suicide in men was higher than in women. Other studies in Iran were consistent with our results^6, 23, 24^. Age is another important factor related to suicide. In this study, higher frequency of suicide was seen in age group 15-29 yr old and middle-aged people, in consistent with another study in Iran^23^.

According to our result, most suicides had occurred via hanging method. Similar result was seen in Rezaeian et al’s^25^. In addition, younger people more frequently select more violet methods for suicide such as firearm but in older people suicide happened via hanging and poisoning, besides, gender, age and educational level were affective variables for selecting the suicide method^6^. Frequency of suicide by self-immolation (36.2%) in Ilam Province was very close to hanging (35.2%). In a study, suicide by self-immolation was the most common method of suicide in this province and the suicide rate was highest among 20–29 yr old. Higher successful suicide rate was found among the married person compared to singles^26^.

Assessment of suicide deaths (frequency) per month in these four provinces showed that suicide was more prevalent in spring and summer. In Iran the highest and the lowest number of successful suicides happened in warm (June-July) and cold seasons (November to January), respectively. Difference between these two seasons was statistically significant^7^_._

Spatial and temporal pattern of suicide was different in these provinces. Suicide rate in all four provinces were more than the national average. Ilam Province had the highest suicide rate in 2015 among four provinces. In Ilam, the highest suicide rate was seen in 2010 (25.0 per 100,000 population) and the lowest rate in 2013 (15.2 per 100,000 population). In Kohgiluyeh and Boyer-Ahmad, the maximum and minimum suicide rate was respectively related to 2006 (16.9 per 100,000 population) and 2009 (4.1 per 100,000 population). The highest suicide rate in Kermanshah was seen in 2013 (27.6 per 100,000 population) and the lowest was observed in 2010 (12.1 per 100,000 population) and the suicide rate in this province was declining over the past three years.

According to Table 1, Kermanshah Province had the highest number of successful suicides among the four provinces during the study period. In Kermanshah, sex, marital, educational and job status were the important risk factors for suicidal death. In addition, suicide in unemployed, homemakers and retired person was higher than others^27^.

Time series plot of Ilam and Lorestan provinces, showed a stationary trend over time. Data series of these two provinces, did not have large fluctuations in variance and mean, also term of seasonality was not observed in these two series. Series of Kermanshah showed stationary trend up to 2012 and since then an increasing trend until 2014 and then showed a decreasing trend. The Kermanshah's proposed model was of multiplicative error and additive seasonality but trend components were not seen. Kohgiluyeh and Boyer-Ahmad showed decreasing trend in three first years of the study with a consistent trend since 2009 to 2013, since then, the fluctuations in the province have been different.

Results of this study showed differences in the rate and trend of suicide in four provinces. This could be due to differences in the properties of population under study, different demographic, cultural, economic conditions and social factors and other factors that affect this matter.

**Strengths and Limitations:** We used data on successful suicide recorded in the Legal Medicine Organization. According to the Iran law all suspicious deaths should be investigated in this organization, hence our data is the most comprehensive data about successful suicides in Iran. However, because suicide is a sensitive issue among population there may be some under reporting by families.

We did not have access to attempted suicides' data, so there was no possibility to compare characteristics of attempted and successful suicides. Better results and more information about this phenomenon could be obtained by analyzing data on both attempted and successful suicides.

## Conclusions

Considering the current situation in these four provinces, it is suggested that intervention measures be designed in order to decrease suicide rate particularly in the age group 15-29 yr, and implemented as a pilot study, especially in these four provinces of Iran. Due to the higher suicide by self-immolation method compared to the Iran averages rate, in Ilam and Lorestan provinces, it is recommended that interventional and prevention measures be taken in high risk persons of these two provinces.

The result of this study highlight the necessity for further studies to detect high risk population for suicide as the first preventing step towards planning a highly organized approach to control and limit the suicide rate. It is also recommended to do time series analysis of suicide in other provinces.

## Acknowledgment

This study is a part of MSc thesis supported by Shahid Beheshti University of Medical Sciences and Legal Medicine Organization of Iran. We thank the Legal Medical Organization and Legal Medicine Research Center and their colleague for providing the suicide data for analyses.

## Conflict of interest statement

The authors declare that have no conflicts of interest.

## Funding

None.

## Highlights

- Suicide trend was different in four studied provinces.
- Suicide rate of all four provinces were more than the national average.
- Hanging in men and self-immolation in women were the most frequent methods of suicide.
